# Treatment outcomes among children, adolescents, and adults on treatment for tuberculosis in two metropolitan municipalities in Gauteng Province, South Africa

**DOI:** 10.1186/s12889-019-7257-4

**Published:** 2019-07-22

**Authors:** Kaitlyn M. Berry, Carly A. Rodriguez, Rebecca H. Berhanu, Nazir Ismail, Lindiwe Mvusi, Lawrence Long, Denise Evans

**Affiliations:** 1 Department of Global Health, Boston University School of Public Health, Boston, MA USA; 2 Department of Epidemiology, Boston University School of Public Health, Boston, MA USA; 3 Health Economics and Epidemiology Research Office, Faculty of Health Sciences, Department of Internal Medicine, School of Clinical Medicine, University of the Witwatersrand, Postnet Suite 212, Private Bag X2600, Houghton, Johannesburg, 2041 South Africa; 4 National Institute for Communicable Diseases, Johannesburg, South Africa; 5 Faculty of Health Sciences, University of Pretoria, Pretoria, South Africa; 6 Faculty of Health Sciences, Department of Internal Medicine, University of Witwatersrand, Johannesburg, South Africa; 7 National Department of Health, Tuberculosis Cluster, Pretoria, South Africa

**Keywords:** Tuberculosis, South Africa, Tuberculosis outcomes, Pediatrics, Adults, Young adults, National electronic TB register (ETR)

## Abstract

**Background:**

Gauteng Province has the second lowest tuberculosis (TB) incidence rate in South Africa but the greatest proportion of TB/HIV co-infection, with 68% of TB patients estimated to have HIV. TB treatment outcomes are well documented at the national and provincial level; however, knowledge gaps remain on how outcomes differ across detailed age groups.

**Methods:**

Using data from South Africa’s National Electronic TB Register (ETR), we assessed all-cause mortality and loss to follow-up (LTFU) among patients initiating treatment for TB between 01/2010 and 12/2015 in the metropolitan municipalities of Ekurhuleni Metropolitan Municipality and the City of Johannesburg in Gauteng Province. We excluded patients who were missing age, had known drug-resistance, or transferred into TB care from sites outside the two metropolitan municipalities. Among patients assigned a treatment outcome, we investigated the association between age group at treatment initiation and mortality or LTFU (treatment interruption of ≥2 months) within 10 months after treatment initiation using Cox proportional hazard models and present hazard ratios and Kaplan-Meier survival curves.

**Results:**

We identified 182,890 children (<10 years), young adolescent (10–14), older adolescent (15–19), young adult (20–24), adult (25–49), and older adult (≥50) TB cases without known drug-resistance. ART coverage among HIV co-infected patients was highest for young adolescents (64.3%) and lowest for young adults (54.0%) compared to other age groups (all over 60%). Treatment success exceeded 80% in all age groups (*n* = 170,017). All-cause mortality increased with age. Compared to adults, young adults had an increased hazard of LTFU (20–24 vs 25–49 years; aHR 1.43 95% CI: 1.33, 1.54) while children, young adolescents, and older adults had lower hazard of LTFU. Patients with HIV on ART had a lower risk of LTFU, but greater risk of death when compared to patients without HIV.

**Conclusions:**

Young adults in urban areas of Gauteng Province experience a disproportionate burden of LTFU and low coverage of ART among co-infected patients. This group should be targeted for interventions aimed at improving clinical outcomes and retention in both TB and HIV care.

## Background

South Africa has the highest incidence of tuberculosis (TB) in the world, with an estimated 781 cases per 100,000 people in 2016 [1]. Gauteng Province, the most populous of the nine provinces in South Africa and home to the major urban center of Johannesburg, has the second lowest TB incidence rate but greatest proportion of TB/HIV co-infection, with 68% of TB patients estimated to have HIV [1]. Despite HIV being a major risk factor for poor TB treatment outcomes [2, 3], Gauteng Province has historically maintained a TB treatment success rate greater than the national average (85% vs 80%) [4].

Knowledge gaps remain regarding how TB treatment outcomes differ by age, especially in countries with high TB/HIV co-infection such as South Africa. Prior studies on TB outcomes have focused on children, adolescents, or adults individually without attention to how TB outcomes differ across the full age spectrum within the same setting. Currently adolescents (10–19 years) are recorded in age groups 5–14 years and 15–24 years, and there is no routine reporting on this specific age group [5]. A thorough comparative analysis of treatment outcomes may help to identify specific age categories that are most vulnerable and should therefore be targeted for interventions or studies to better understand predictors of poor outcomes.

Young adolescents (10–14 years), older adolescents (15-19 years) and young adults (20–24 years) represent a particularly vulnerable population with unique needs for TB management [5]. While transitioning from childhood to adulthood, adolescents establish patterns of behavior and lifestyle choices that affect both their current and future health [6]. In general, adolescents are reported to have poorer adherence to treatment when compared to adults, whereas for children this depends on the care practices of the parents [7, 8]. Available evidence on TB treatment outcomes in these populations are conflicting. Some studies have reported adolescents (10–19 years) have better treatment outcomes, less death, and less severe indicators and co-morbidities than adults (>25 years) [9], while others report that older adolescents (15–19 years) have high rates of default and treatment failure [10]. Older adolescents and young adults with TB-HIV co-infection have poorer outcomes and are at a high risk of TB treatment discontinuation [11]. The consequences of inadequate and incomplete TB treatment are serious: prolonged illness and disability for the patient, infectiousness of the patient causing continued TB transmission in the community, development of drug resistant TB, and the possibility of death [3]. Considering adult-type pulmonary TB (PTB) is common among adolescents and is often diagnosed late, adolescents can pose a significant transmission risk to the community [12].

Since there is a lack of evidence on how TB outcomes differ across the full age spectrum within the same setting, this study aims to describe treatment outcomes among children, adolescents, young adults, adults, and older adults on treatment for TB in two metropolitan municipalities in Gauteng Province, South Africa.

## Methods

### Data source

In 2005, the national Electronic TB Register (ETR) was implemented in South Africa to monitor indicators essential to understanding the burden and management of TB [13]. Along with key TB indicators, ETR captures basic information on HIV status and HIV-related treatment for TB patients. Health facilities enter patient information into two paper registers: one for presumptive TB cases for those who present with symptoms suggestive of TB and a second with key demographic and clinical information on all persons diagnosed with TB disease who initiate treatment (TB register). Laboratory results from the National Health Laboratory Services (NHLS) are sent to the health facility which manually enters the result in the patient’s primary medical record, known as the “TB blue card”. Presumptive cases who test positive for TB are subsequently recorded in the TB treatment register [13]. The TB registers are sent to the sub-district office, where the information is entered into ETR. Facility level data is aggregated into sub-district and district level for use by the National TB Program (NTP). Finally, the NTP quantifies, monitors, and evaluates data on the TB burden and treatment outcomes.

Our analysis was restricted to ETR data collected between January 2010 and December 2015 in the metropolitan municipalities of the City of Johannesburg and Ekurhuleni Metropolitan Municipality, located in Gauteng Province. The Ekurhuleni Metropolitan Municipality is situated in the Eastern part of Gauteng Province, about 20 km from the largest city in South Africa, Johannesburg, located in the City of Johannesburg Metropolitan Municipality. Both municipalities are highly urbanized, with the majority living in urban settlements ranging from informal settlements to residential suburbs.

### Exclusions

We excluded patients who were entered into ETR but did not have TB (e.g. those who had received isoniazid [INH] prophylaxis/INH preventive therapy [IPT]). We also excluded those who had missing age information and those who transferred into TB care from sites outside the two metropolitan municipalities because we did not have information on their treatment history (i.e. baseline characteristics such as smear status, diagnosis method, etc.). In addition, patients were excluded if they had evidence of drug resistance. Starting in 2013, there was national coverage of Xpert MTB/RIF (Cepheid, USA) resulting in universal testing for rifampicin (RIF) resistance in patients who had a positive Xpert MTB/RIF test [3]. Further testing for INH or second-line TB drug susceptibility is not routinely performed in RIF susceptible patients. Prior to 2013, drug susceptibility testing (DST) was only performed for retreatment cases, individuals who failed to smear convert after 2 months of intensive treatment, in cases of treatment failure, and in close contacts of people with known drug resistance [14]. Consistent with prior work [15], our analyses of TB outcomes further excluded patients who were still on treatment and patients who transferred out or for whom no treatment outcome had been assigned.

### Measures

We categorized patients into children (<10 years), young adolescents (10–14 years) [14, 16], older adolescents (15–19 years) [17, 18], young adults (20–24 years) [18], adults (25–49 years), and older adults (≥50 years) [19]. Additional demographic information included district within Gauteng (Ekurhuleni Metropolitan Municipality, City of Johannesburg) and sex (male, female).

Patients were classified as bacteriologically confirmed if they were diagnosed through Xpert MTB/RIF (Cepheid, USA), smear microscopy or culture. If aspiration/biopsy or cerebral spinal fluid (CSF) was listed, the case was considered bacteriologically confirmed although the corresponding laboratory method was unknown. Patients diagnosed via X-ray or tuberculin skin test (TST) were classified as clinically diagnosed, while those missing diagnostic information were considered to have an unknown case definition. Patients were also categorized as new, previously treated (relapse, retreatment after failure, retreatment after loss to follow-up/default), or unknown previous TB treatment history [3].

During the study period, care was provided according to the South African national TB treatment guidelines [3]. In adults, the standard fixed dose regimen (regimen 1) comprised a two month long intensive phase of treatment with rifampicin (R), isoniazid (H), pyrazinamide (Z), and ethambutol (E) (2 RHZE) followed by a four month continuation phase of daily R and H (4 RH) [3, 14]. Prior to 2013 before the retreatment regimen was discontinued [3], TB patients who required retreatment (e.g. due to treatment interruption or recurrence of disease) would have received the retreatment regimen containing streptomycin (S) in addition to RHZE over a longer duration (regimen 2; 2HRZES/1HRZE/5HRE). Children receive the same standard fixed dose regimen (regimen 1), except that the dosage (mg/day) is reduced, or a regimen of only RHE.

Additional TB treatment information included smear status at treatment initiation (positive, negative or missing), registration year (2010–2015), classification of disease (pulmonary TB, extra-pulmonary TB), cotrimoxazole prophylaxis (yes, no, missing/unknown), intensive phase directly observed treatment short-course (DOTS) supervision (yes, no, unknown/missing) and continuation phase DOTS supervision (yes, no, unknown/missing).

HIV status was categorized into HIV positive on ART, HIV positive not on ART, HIV positive with unknown ART status, HIV negative, and HIV status unknown. We report whether ART was initiated prior to or after TB treatment starting in 2014 when ETR began to capture ART start date. We also reported the CD4 count (cells/mm^3^) for HIV positive patients where available.

Sputum smear conversion was defined as two consecutive negative smears at least 30 days apart among those who were smear positive at baseline. We assessed smear conversion within two months of treatment initiation in addition to smear conversion at any time point during treatment.

Treatment outcomes were defined as cured, completed, failed, lost to follow-up, all-cause mortality, or transferred out (Table 1) in accordance with WHO definitions and reporting framework for TB [20]. Treatment duration typically lasts 6 months for pulmonary TB while extra-pulmonary TB cases have their own defined duration in accordance with national guideline [3]. In some instances, the treatment duration may be extended. Reasons include: 1) the intensive phase could be extended to 3 months in the absence of smear conversion, defined as remaining AFB+ after 2 months of treatment, 2) the continuation phase could be extended to 9 months in the event of severe or complicated disease, 3) if a treatment interruption occurred lasting <2 months, treatment could be extended by the number of days that the patient did not take treatment without restarting treatment from the beginning and 4) when the retreatment regimen 2 (2HRZES/1HRZE/5HRE) was still being used (prior to 2013), the duration of treatment for previously treated cases was 8 months instead of 6 months for new pulmonary TB cases [3, 14]. Since the majority (98%) of patients have a treatment outcome assigned within 10 months after treatment initiation we opted to report treatment outcomes at this point.

**Table 1:** Treatment Outcomes for Tuberculosis [17]

Outcome	Definition
Cure	Patient whose baseline smear (or culture) was positive at the beginning of the treatment and is smear / culture negative in the last month of treatment and on at least one previous occasion at least 30 days prior.
Treatment completed	Patient whose baseline smear (or culture) was positive at the beginning and has completed treatment but does not have a negative smear / culture in the last month of treatment and on at least one previous occasion more than 30 days prior. The smear examination may not have been done or the results may not be available at the end of treatment.
Treatment failure	Patient whose baseline smear (or culture) was positive and remains or becomes positive again at 5 months or later during treatment. This definition excludes those patients who are diagnosed with rifampicin resistant or multi-drug resistant TB.
Died	A patient who dies for any reason during the course of treatment.
Lost to follow-up	A patient whose treatment was interrupted for two consecutive months or more.
Not evaluated	A patient for whom no treatment outcome is assigned. (This includes cases “transferred out” to another treatment unit and whose treatment outcome is unknown).
Treatment Success	The sum of cured and treatment completed.

### Statistical analysis

We summarized demographic and treatment information by age group using proportions for categorical variables and median and interquartile range (IQR) for continuous variables. Treatment outcomes are described as reported in ETR, within 10 months after treatment initiation, by age group.

The primary outcomes of interest included all-cause mortality and loss to follow-up (treatment interruption of ≥2 months). Patients were followed from the start of TB treatment until the earliest of death, loss to follow-up, or outcome within 10 months after TB treatment initiation.

We investigated the association between age group (at start of TB treatment) and all-cause mortality and loss to follow-up using Cox proportional hazard models. Variables with a *p*-value less than 0.25 in the univariate analysis along with a priori variables (e.g. age, sex, disease classification, HIV status) were included in the final multivariate model. We present the hazard ratio and corresponding 95% confidence interval. We used Kaplan-Meier survival curves to display survival probabilities from TB treatment initiation within 10 months of follow-up for both all-cause mortality and loss to follow-up.

Sensitivity analysis included all-cause mortality and loss to follow-up among all patients who had a treatment outcome assigned in ETR, regardless of when the outcome occurred. We also used the method of Fine and Grey, competing risks regression as an alternative to Cox regression for time to event data in the presence of a competing risk (e.g. death) [21–23].

We used STATA Version 15 (StataCorp, Texas, USA) for all analyses. Ethics approval for this study was granted by the Human Research Ethics Committee (Medical) of the University of the Witwatersrand, Johannesburg (protocol M160971). The study was a retrospective review of programmatic data and a waiver of informed consent was granted to retrospectively review these records.

## Results

**Fig. 1:**
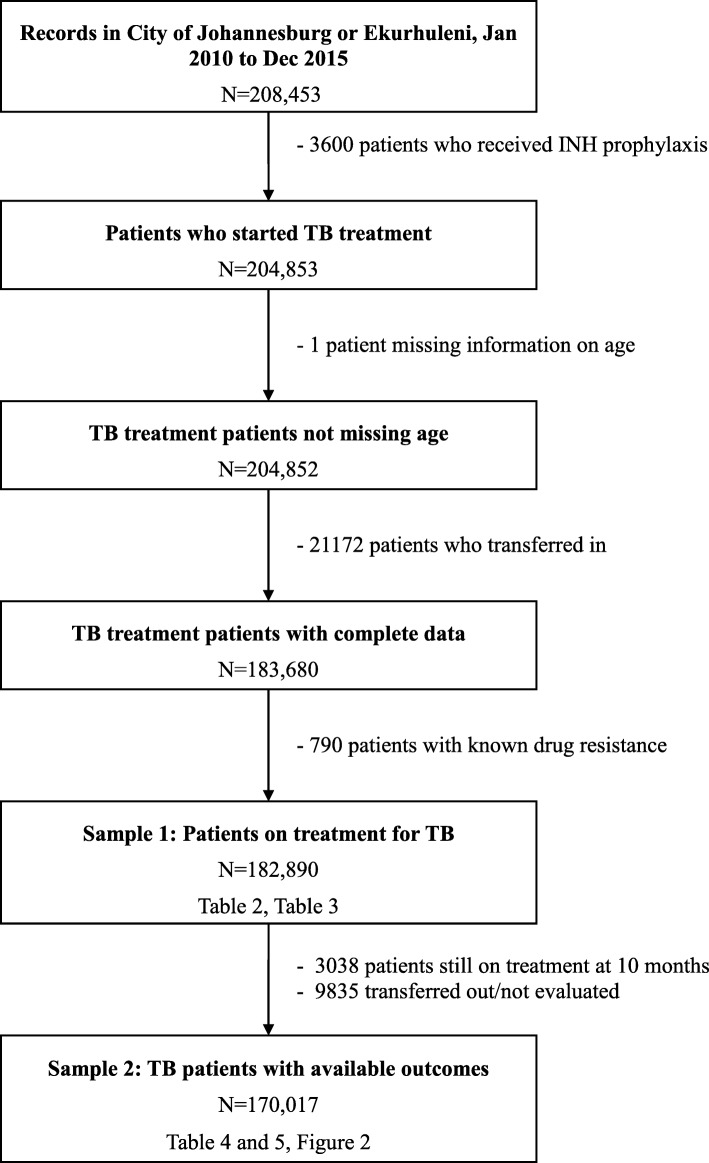
Study inclusion/exclusion criteria

We identified 208,453 ETR records in the City of Johannesburg and Ekurhuleni Metropolitan Municipality between January 2010 and December 2015. Of these patients, 25,563 were not eligible for our study, leaving a sample of 182,890 children, adolescent, young adult, adult, and older adult TB cases (Fig. 1). The sample represented 53.2% (*n* = 343,954) and 6.7% (*n* = 2,738,481) of the total number of all cases reported in ETR for Gauteng Province and South Africa, respectively during the same period, was predominately from the City of Johannesburg (64.9%) and cases were distributed evenly across registration year (Table 2).

**Table 2:** Characteristics of cases treated for TB disease by age group (*n* = 182,890)

Characteristics		All *N* = 182890	Children (<10 years) *N* = 18259	Young Adolescents (10–14 years) *N* = 2032	Older Adolescents (15–19 years) *N* = 3931	Young Adults (20–24 years) *N* = 11686	Adults (25–49 years) *N* = 123363	Older Adults (≥50 years) *N* = 23619
District in Gauteng	Ekurhuleni Metropolitan Municipality	64304 (35.16%)	6110 (33.46%)	832 (40.94%)	1585 (40.32%)	4310 (36.88%)	42575 (34.51%)	8892 (37.65%)
	City of Johannesburg	118586 (64.85%)	12149 (66.54%)	1200 (59.06%)	2346 (59.68%)	7376 (63.12%)	80788 (65.49%)	14727 (62.35%)
Registration year	2010	32704 (17.88%)	3126 (17.12%)	367 (18.06%)	726 (18.47%)	2322 (19.87%)	23333 (18.10%)	3830 (16.22%)
	2011	31999 (17.50%)	3460 (18.95%)	393 (19.34%)	676 (17.20%)	2108 (18.04%)	21508 (17.43%)	3854 (16.32%)
	2012	30844 (16.86%)	3258 (17.84%)	400 (19.69%)	672 (17.09%)	1927 (16.49%)	20822 (16.88%)	3765 (15.94%)
	2013	30872 (16.88%)	3302 (18.08%)	310 (15.26%)	659 (16.76%)	1891 (16.18%)	20608 (16.71%)	4102 (17.37%)
	2014	29506 (16.13%)	2585 (14.16%)	264 (12.99%)	597 (15.19%)	1857 (15.89%)	20065 (16.27%)	4138 (17.52%)
	2015	26965 (14.74%)	2528 (13.85%)	298 (14.67%)	601 (15.29%)	1581 (13.53%)	18027 (14.61%)	3930 (16.64%)
Sex	Female	82341 (45.02%)	8875 (48.61%)	1069 (52.61%)	2379 (60.52%)	7070 (60.50%)	54312 (44.03%)	8636 (36.56%)
Case definition^a^	Bacteriologically confirmed	126360 (69.09%)	1265 (6.93%)	1130 (55.61%)	3086 (78.50%)	9247 (79.13%)	94042 (76.23%)	17590 (74.47%)
	Clinically diagnosed	29658 (16.22%)	9309 (50.98%)	467 (22.98%)	418 (10.63%)	1224 (10.47%)	15126 (12.26%)	3114 (13.18%)
	Missing/Unknown	26872 (14.69%)	7685 (42.09%)	435 (21.41%)	427 (10.86%)	1215 (10.40%)	14195 (11.51%)	2915 (12.34%)
Full case definition	Xpert MTB/RIF	28482 (15.57%)	239 (1.31%)	188 (9.25%)	694 (17.65%)	1929 (16.51%)	20959 (16.99%)	4473 (18.94%)
	Smear microscopy	87192 (47.67%)	763 (4.18%)	810 (39.86%)	2195 (55.84%)	6642 (56.84%)	65102 (52.77%)	11680 (49.45%)
	Culture	5638 (3.08%)	45 (0.25%)	53 (2.61%)	97 (2.47%)	356 (3.05%)	4282 (3.47%)	805 (3.41%)
	Aspiration/biopsy, lab method unknown	3923 (2.15%)	146 (0.80%)	60 (2.95%)	80 (2.04%)	251 (2.15%)	2901 (2.35%)	485 (2.05%)
	CSF, lab method unknown	1125 (0.62%)	72 (0.39%)	19 (0.94%)	20 (0.51%)	69 (0.59%)	798 (0.65%)	147 (0.62%)
	X-ray	23720 (12.97%)	3600 (19.72%)	398 (19.59%)	411 (10.46%)	1213 (10.38%)	15033 (12.16%)	3095 (13.10%)
	Tuberculin skin test (TST)	5938 (3.25%)	5709 (31.27%)	69 (3.40%)	7 (0.18%)	11 (0.09%)	123 (0.10%)	19 (0.08%)
	Missing/Unknown	26872 (14.69%)	7685 (42.09%)	435 (21.41%)	427 (10.86%)	1215 (10.40%)	14195 (11.51%)	2915 (12.34%)
Cases diagnosed with Xpert MTB/RIF by year	2010	0 (0.00%)	0 (0.00%)	0 (0.00%)	0 (0.00%)	0 (0.00%)	0 (0.00%)	0 (0.00%)
	2011	8 (0.03%)	0 (0.00%)	0 (0.00%)	0 (0.00%)	1 (0.05%)	7 (0.03%)	0 (0.00%)
	2012	366 (1.19%)	2 (0.06%)	1 (0.25%)	7 (1.045)	22 (1.14%)	291 (1.40%)	43 (1.14%)
	2013	2136 (6.92%)	11 (0.335)	8 (2.58%)	49 (7.44%)	130 (6.87%)	1603 (7.78%)	335 (8.17%)
	2014	8992 (30.48%)	81 (3.13%)	56 (21.21%)	213 (35.68%)	629 (33.87%)	6618 (32.98%)	1395 (33.71%)
	2015	16980 (62.97%)	145 (5.74%)	123 (41.28%)	425 (70.72%)	1147 (72.55%)	12440 (69.01%)	2700 (68.70%)
Patient category	New Patient	172252 (94.18%)	18058 (98.90%)	1957 (96.31%)	3776 (96.06%)	11125 (95.20%)	115272 (93.42%)	22064 (93.42%)
	Previously treated	7459	159	47	113	435	5650	1055
	Relapse	5227 (2.86%)	113 (0.62%)	32 (1.57%)	66 (1.68%)	233 (1.99%)	3993 (3.24%)	790 (3.34%)
	Re-treatment after failure	810 (0.44%)	14 (0.08%)	2 (0.10%)	18 (0.46%)	43 (0.37%)	612 (0.50%)	121 (0.51%)
	Re-treatment after LTFU	1422 (0.78%)	32 (0.18%)	13 (0.64%)	29 (0.74%)	159 (1.36%)	1045 (0.85%)	144 (0.61%)
	Unknown previous TB treatment history	3179 (1.74%)	42 (0.23%)	28 (1.38%)	42 (1.07%)	126 (1.08%)	2441 (1.98%)	500 (2.12%)
Classification of Disease	Pulmonary TB	15270 (83.50%)	17474 (95.70%)	1606 (79.04%)	3321 (84.48%)	9831 (84.13%)	100842 (81.74%)	19646 (83.18%)
	EPTB	30170 (16.50%)	785 (4.30%)	426 (20.96%)	610 (15.52%)	1855 (15.87%)	22521 (18.26%)	3973 (16.82%)
Smear status at initiation	Positive	68282 (37.34%)	301 (1.65%)	445 (21.90%)	2003 (50.95%)	5961 (51.01%)	50764 (41.15%)	8808 (37.29%)
	Negative	43808 (23.95%)	612 (3.35%)	518 (25.49%)	798 (20.30%)	2429 (20.79%)	32827 (26.61%)	6624 (28.05%)
	Missing	70800 (38.71%)	17346 (95.00%)	1069 (52.61%)	1130 (28.75%)	3296 (28.20%)	39772 (32.24%)	8187 (34.66%)
HIV status	Total Positive	118326	3325	1000	1386	5711	93818	13086
	Positive, on ART	71421 (39.05%)	2016 (11.04%)	643 (31.64%)	853 (21.70%)	3084 (26.39%)	56637 (45.91%)	8188 (34.67%)
	Started prior to TB treatment^b^	215 (0.63%)	1 (0.12%)	4 (1.39%)	3 (0.68%)	9 (0.62%)	161 (0.60%)	37 (0.88%)
	Started with/after TB treatment^b^	631 (1.85%)	7 (0.83%)	1 (0.35%)	2 (0.46%)	28 (0.46%)	510 (1.89%)	83 (1.96%)
	Start date unknown^b^	33346 (97.53%)	837 (99.05%)	283 (98.26%)	433 (98.86%)	1409 (97.44%)	26276 (97.51%)	4108 (97.16%)
	Positive, not on ART	29757 (16.27%)	693 (3.80%)	187 (9.20%)	323 (8.22%)	1682 (14.39%)	23784 (19.28%)	3088 (13.07%)
	Positive, ART status unknown	17148 (9.38%)	616 (3.37%)	170 (8.37%)	210 (5.34%)	945 (8.09%)	13397 (10.86%)	1810 (7.66%)
	Negative	40784 (22.30%)	10397 (56.94%)	668 (32.87%)	1859 (47.29%)	4283 (36.65%)	16468 (13.35%)	7109 (30.10%)
	HIV status unknown	23780 (13.00%)	4537 (24.85%)	364 (17.91%)	686 (17.45%)	1692 (14.48%)	13077 (10.60%)	3424 (14.50%)
CD4 count (cells/mm^3^)^c^	≤100	26013 (21.98%)	279 (8.39%)	155 (15.50%)	247 (17.82%)	1056 (18.49%)	21790 (23.23%)	2486 (19.00%)
	101–250	20433 (17.27%)	282 (8.48%)	112 (11.20%)	219 (15.80%)	1035 (18.12%)	16615 (17.71%)	2170 (16.58%)
	251–350	6685 (5.65%)	109 (3.28%)	67 (6.70%)	87 (6.28%)	407 (7.13%)	5285 (5.63%)	730 (5.58%)
	351–500	4877 (4.12%)	129 (3.88%)	49 (4.90%)	61 (4.40%)	349 (6.11%)	3765 (4.01%)	524 (4.00%)
	>500	3304 (2.79%)	256 (7.70%)	42 (4.20%)	59 (4.26%)	247 (4.32%)	2311 (2.46%)	389 (2.97%)
	Missing	57014 (48.18%)	2270 (68.27%)	575 (57.50%)	713 (51.44%)	2617 (45.82%)	44052 (46.95%)	6787 (51.86%)
	Median (IQR)	125 (51–248)	226 (90–494)	160 (42–328)	160 (54–300)	164 (69–306)	120 (50–237)	134 (59–259)
Started on cotrimoxazole prophylaxis^c^	Yes	93554 (79.06%)	2452 (73.74%)	767 (76.70%)	1078 (77.78%)	4319 (75.63%)	74543 (79.45%)	10395 (79.44%)
	No	14772 (12.48%)	468 (14.08%)	132 (13.20%)	184 (13.28%)	824 (14.43%)	11535 (12.30%)	1629 (12.45%)
	Missing/unknown	10000 (8.45%)	405 (12.18%)	101 (10.10%)	124 (8.95%)	568 (9.95%)	7740 (8.25%)	1062 (8.12%)
Treatment regimen	Regimen 1 (2 RHZE/4 RH)	157206 (85.96%)	2015 (11.04%)	1880 (92.52%)	3788 (96.36%)	11160 (95.50%)	116128 (94.14%)	22235 (94.14%)
	Regimen 2 (2 RHZES/1 HRZE/5 HRE)	9364 (5.12%)	55 (0.30%)	68 (3.35%)	139 (3.54%)	523 (4.48%)	7198 (5.83%)	1381 (5.85%)
	Regimen 3 (2 RHZE / 4 RH)^d^	16312 (8.92%)	16185 (88.64%)	83 (4.08%)	3 (0.08%)	3 (0.03%)	35 (0.03%)	3 (0.01%)
	Other	8 (0.00%)	4 (0.02%)	1 (0.05%)	1 (0.03%)	0	2 (0.00%)	0
Treatment supervision (Intensive Phase)	Yes	29170 (15.95%)	1708 (14.38%)	302 (14.86%)	622 (15.82%)	2012 (17.22%)	20277 (16.44%)	3249 (13.76%)
	No	129116 (70.60%)	13478 (73.82%)	1457 (71.70%)	2740 (69.70%)	8143 (69.68%)	86550 (70.16%)	16748 (70.91%)
	Missing	24604 (13.45%)	2073 (11.35%)	273 (13.44%)	569 (14.47%)	1531 (13.10%)	16536 (13.40%)	3622 (15.34%)
Treatment supervision (Continuation Phase)	Yes	7722 (4.22%)	946 (5.18%)	104 (5.12%)	157 (3.99%)	511 (4.37%)	5211 (4.22%)	793 (3.36%)
	No	150564 (82.32%)	15240 (83.47%)	1655 (81.45%)	3205 (81.53%)	9644 (82.53%)	101616 (82.37%)	19204 (81.31%)
	Missing	24604 (13.45%)	2073 (11.35%)	273 (13.44%)	569 (14.47%)	1531 (13.10%)	16536 (13.40%)	3622 (15.34%)
Smear conversion at 2 months^e^	Yes	22476 (68.66%)	84 (64.12%)	163 (71.49%)	706 (68.08%)	2055 (70.09%)	16725 (68.91%)	2743 (66.27%)
	No	10261 (31.34%)	47 (35.88%)	65 (28.51%)	331 (31.92%)	877 (29.91%)	7545 (31.09%)	1396 (33.73%)
	N/A (Not smear positive at baseline or < 2 available smears)	150153	18128	1804	2894	8754	99093	19480
Any smear conversion^f^	Yes	56628 (93.3%)	251 (92.62%)	393 (96.56%)	1728 (92.37%)	4918 (92.61%	42105 (93.36%)	7778 (92.99%
	No	4069 (6.70%)	20 (7.38%)	14 (3.44%)	103 (5.63%)	391 (7.36%)	2996 (6.64%)	545 (7.01%)
	N/A (Not smear positive at baseline or < 2 available smears)	122193	17988	1625	2100	6377	78262	15841

*Xpert MTB/RIF* GeneXpert MTB/RIF, *CSF* cerebral spinal fluid, *EPTB* extra-pulmonary tuberculosis, *LTFU* loss to follow-up, *HIV* human immunodeficiency virus, *ART* antiretroviral therapy, *IQR* interquartile range, *S* streptomycin, *R* rifampicin, *H* isoniazid, *Z* pyrazinamide, *E* ethambutol

^a^Bacteriologically confirmed includes Xpert MTB/RIF, smear, or culture (and if aspiration/biopsy or cerebral spinal fluid (CSF) was listed, although the corresponding laboratory method was unknown). Clinically diagnosed includes X-ray and tuberculin skin test

^b^ART start date only reported for patients who initiated TB treatment in 2014 or 2015

^c^Reported for HIV positive patients only

^d^Regimen 3, for children <8 years and <30 kg with complicated TB disease, is the same as Regimen 1 except that the dosage (mg/day) is reduced

^e^Smear conversion at two months is two consecutive negative smears at least 30 days apart within two months of treatment initiation. One smear is sent monthly for smear microscopy

^f^Any smear conversion is two consecutive negative smears at least 30 days apart at any time after treatment initiation. Ascertained at the end of treatment

Although the sample was 45% female overall, the older adolescent and young adult age groups were disproportionately female (60.5% for both groups). The majority of cases were diagnosed with pulmonary TB (83.5%), were new cases (94.2%), and were bacteriologically confirmed (69.1%). Bacteriologic confirmation was low among children (6.9%) and young adolescents (52.6%) and over 70% for older adolescents, young adults, adults, and older adults. Smear microscopy was the most common method of diagnosis in the overall sample (47.7%). The proportion of cases diagnosed by Xpert MTB/RIF was 15.6% overall but increased from 0% in 2010 to 63.0% by the end of the study period. In 2015, diagnosis by Xpert remained low for children (5.7%) and young adolescents (41.3%) but was common for older adolescents (70.7%), young adults (72.6%), adults (69.0%), and older adults (68.7%).

Two-thirds (64.7%) of the TB cases were co-infected with HIV. Co-infection was highest among adults (76.1%) followed by older adults (55.4%), young adolescents (49.2%), young adults (48.9%), older adolescents (35.3%), and children (18.2%). Overall, 60.4% of HIV positive cases (71421/118326) were on ART while 25.1% were not and 14.5% had an unknown status. ART coverage patients was highest for young adolescents (64.3%) and lowest for young adults (54.0%) compared to other age groups (all over 60%).

Among cases that were smear positive at treatment initiation and had at least two available smear results within the relevant time period, a majority (93.3%; 56628/60697) achieved smear conversion by the end of follow up and approximately two-thirds (68.7%; 22476/32737) achieved smear conversion by two months.

By 10 months of follow-up, 51.5% of all TB cases (*n* = 182,890) completed treatment, 30.4% were cured, 5.4% had died, 5.5% were lost to follow-up, and 0.2% experienced treatment failure (Table 3). The remaining 7.0% were still on treatment or transferred out. The proportion of patients with a successful outcome (cured or treatment completion) declined with age, with the greatest decreases observed between the <10 (91.6%), 10–14 (90.8%), 15–19 (86.5%), and 20–24 (82.0%) age groups.

**Table 3:** Treatment outcomes by within 10 months after treatment initiation (*n* = 182,890)

	All *N* = 182890	Children (<10 years) *N* = 18259	Young Adolescents (10–14 years) *N* = 2032	Older Adolescents (15–19 years) *N* = 3931	Young Adults (20–24 years) *N* = 11686	Adults (25–49 years) *N* = 123363	Older Adults (≥50 years) *N* = 23619
Treatment Success^a^	152672 (83.48%)	16727 (91.61%)	1844 (90.75%)	3399 (86.47%)	9587 (82.04%)	102192 (82.84%)	18923 (80.12%)
All Outcomes							
Completed	94118 (51.46%)	16298 (89.26%)	1410 (69.39%)	1639 (41.69%)	4589 (39.27%)	58757 (47.63%)	11425 (48.37%)
Cured	55625 (30.41%)	244 (1.34%)	390 (19.19%)	1702 (43.30%)	4844 (41.45%)	41351 (33.52%)	7094 (30.04%)
Died	9909 (5.42%)	209 (1.14%)	52 (2.56%)	109 (2.77%)	389 (3.33%)	6713 (5.44%)	2437 (10.32%)
Loss to follow-up (LTFU)	10001 (5.47%)	631 (3.46%)	60 (2.95%)	224 (5.70%)	890 (7.62%)	7046 (5.71%)	1150 (4.57%)
Treatment failed	364 (0.20%)	2 (0.01%)	0 (0.00%)	7 (0.18%)	29 (0.25%)	270 (0.22%)	56 (0.24%)
Transferred out/not evaluated	9835 (5.38%)	681 (3.73%)	75 (3.69%)	191 (4.86%)	782 (6.69%)	7069 (5.73%)	1037 (4.39%)
Still on treatment ≥10 months	3038 (1.66%)	194 (1.06%)	45 (2.21%)	59 (1.50%)	163 (1.39%)	2157 (1.57%)	420 (1.78%)
Person-time in months							
Full sample, median (IQR)	6.1 (6.0–6.9)	6.1 (6.0–6.4)	6.2 (6.0–7.4)	6.1 (6.0–6.8)	6.1 (5.9–6.8)	6.1 (5.9–7.1)	6.1 (5.8–7.0)
Outcome sample, median (IQR) ^b^	6.1 (6.0–6.9)	6.1 (6.0–6.4)	6.2 (6.0–7.3)	6.1 (6.0–6.8)	6.1 (6.0–6.9)	6.2 (6.0–7.1)	6.1 (6.0–6.9)

*IQR* interquartile range

^a^Treatment success is the sum of completed and cured. ^b^Person time calculated among those with a treatment outcomes assigned (i.e. excludes patients still on treatment and patients with no available outcome)

**Fig. 2:**
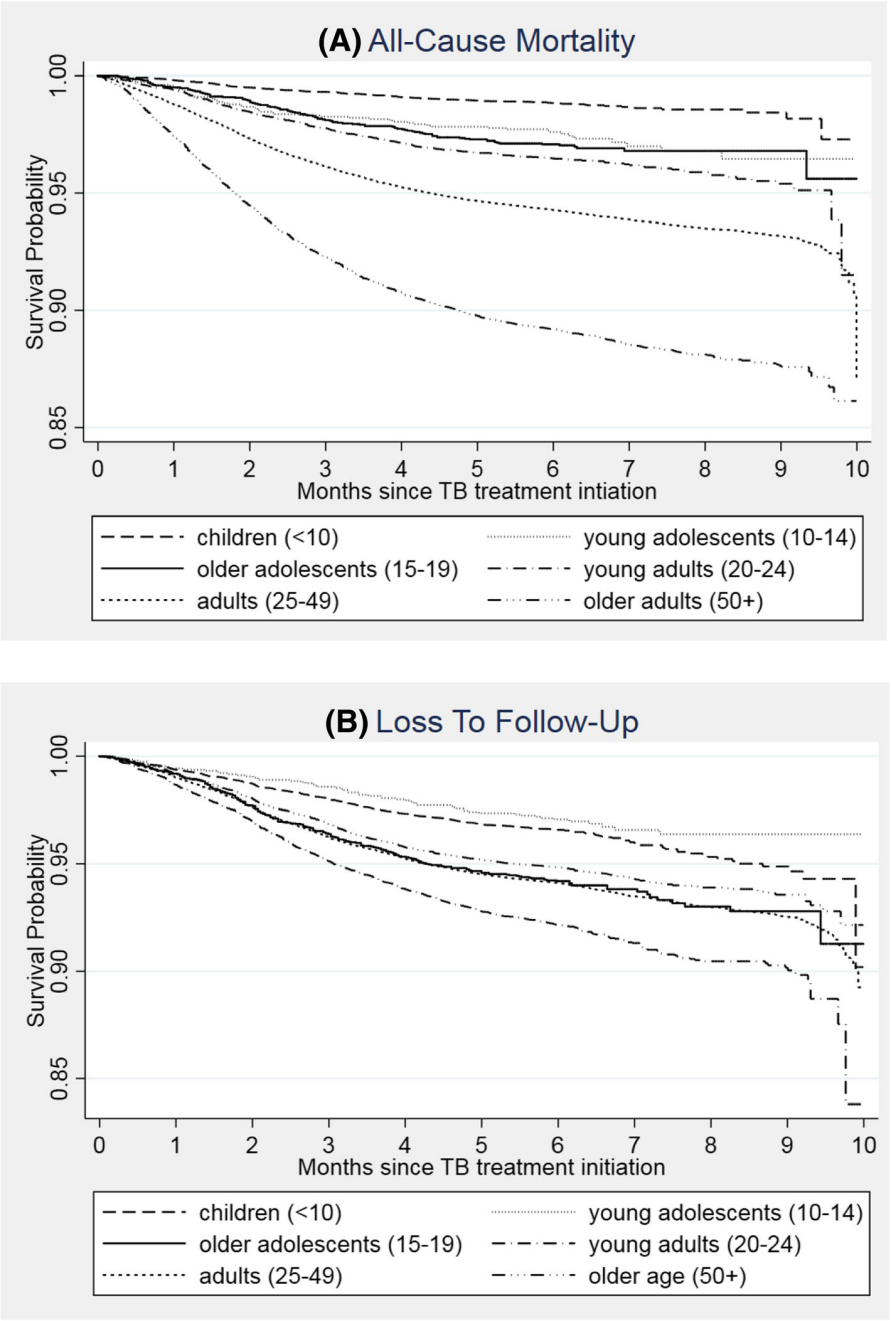
Kaplan Meier survival estimates for (**a**) all-cause mortality and (**b**) loss to follow-up after TB treatment initiation (*n* = 170,017)

Among cases with a treatment outcome within 10 months (*n* = 170,017), 5.8% had died and 5.9% were lost to follow-up (Table 4). Death was similar across sexes (5.9% for women vs 5.8% for men); however, we observed significantly less loss to follow-up among women than men (aHR 0.77, 95% CI: 0.74, 0.81). The proportion of deaths increased with age from 1.2% among children to 11.0% among older adults. Figure 2a shows the probability of survival within 10 months, by age group. Patients younger than 25 years had lower hazard of death than adults aged 25 to 49 while older adults had significantly elevated hazard of death (aHR 2.13, 95% CI 2.03, 2.23).

**Table 4:** Cox proportional hazard models for all-cause mortality and loss to follow-up (*n* = 170,017^a^)

Characteristics		Proportion with outcome [Death]	Crude HR and 95% CI	Adjusted HR and 95% CI	Proportion with outcome [LTFU]	Crude HR and 95% CI	Adjusted HR and 95% CI	Adjusted sub-HR^b^ and 95% CI
Age at start of treatment (years)	<10 (children)	209 (1.20%)	0.20 (0.18–0.23)	0.23 (0.20–0.26)	631 (3.63%)	0.59 (0.54–0.64)	0.62 (0.56–0.68)	0.64 (0.58–0.70)
	10–14 (young adolescents)	52 (2.72%)	0.45 (0.34–0.59)	0.48 (0.37–0.63)	60 (3.14%)	0.49 (0.38–0.63)	0.53 (0.41–0.68)	0.54 (0.42–0.69)
	15–19 (older adolescents)	109 (2.96%)	0.50 (0.41–0.60)	0.62 (0.51–0.75)	224 (6.09%)	0.98 (0.86–1.12)	1.05 (0.92–1.21)	1.07 (0.93–1.22)
	20–24 (young adults)	389 (3.62%)	0.62 (0.56–0.68)	0.71 (0.64–0.78)	890 (8.29%)	1.35 (1.26–1.45)	1.43 (1.33–1.54)	1.44 (1.34–1.55)
	25–49 (adults)	6713 (5.88%)	1.00	1.00	7046 (6.17%)	1.00	1.00	1.00
	≥50 (older adults)	2437 (11.00%)	1.92 (1.84–2.02)	2.13 (2.03–2.23)	1150 (5.19%)	0.87 (0.82–0.92)	0.85 (0.80–0.91)	0.82 (0.77–0.87)
Registration year	2010	1846 (6.26%)	1.01 (0.95–1.08)	0.98 (0.92–1.05)	1844 (6.25%)	1.12 (1.04–1.19)	1.04 (0.97–1.11)	1.04 (0.97–1.12)
	2011	1832 (6.17%)	1.00	1.00	1660 (5.59%)	1.00	1.00	1.00
	2012	1602 (5.57%)	0.91 (0.85–0.97)	0.91 (0.85–0.98)	1670 (5.81%)	1.05 (0.98–1.12)	1.08 (1.01–1.16)	1.08 (1.01–1.16)
	2013	1644 (5.71%)	0.93 (0.87–1.00)	0.96 (0.90–1.03)	1730 (6.01%)	1.09 (1.02–1.16)	1.18 (1.10–1.30)	1.18 (1.10–1.27)
	2014	1540 (5.57%)	0.92 (0.86–0.98)	0.99 (0.92–1.07)	1595 (5.77%)	1.05 (0.98–1.13)	1.21 (1.12–1.30)	1.20 (1.11–1.30)
	2015	1445 (5.63%)	0.93 (0.86–0.99)	1.05 (0.96–1.15)	1502 (5.86%)	1.07 (1.00–1.14)	1.24 (1.13–1.35)	1.23 (1.12–1.34)
Sex	Female	4503 (5.89%)	1.01 (0.97–1.05)	1.04 (1.00–1.08)	3894 (5.10%)	0.77 (0.74–0.81)	0.77 (0.74–0.81)	0.77 (0.74–0.80)
	Male	5406 (5.77%)	1.00	1.00	6107 (6.52%)	1.00	1.00	1.00
Case definition^c^	Bacteriologically confirmed	6556 (5.57%)	1.00	1.00	7130 (6.06%)	1.00	1.00	1.00
	Clinically diagnosed	1704 (6.19%)	1.09 (1.04–1.15)	1.36 (1.28–1.43)	1508 (5.47%)	0.88 (0.84–0.93)	1.02 (0.97–1.09)	1.01 (0.95–1.07)
	Missing/unknown	1649 (6.64%)	1.17 (1.11–1.24)	1.52 (1.44–1.61)	1363 (5.49%)	0.89 (0.84–0.94)	1.04 (0.98–1.11)	1.02 (0.96–1.09)
Patient category	New Patient	9118 (5.68%)	1.00	1.00	9022 (5.62%)	1.00	1.00	1.00
	Previously treated	513 (7.69%)	1.30 (1.19–1.42)	1.23 (1.13–1.35)	701 (10.51%)	1.75 (1.62–1.89)	1.74 (1.61–1.88)	1.74 (1.61–1.88)
	Unknown TB treatment history	278 (9.80%)	1.64 (1.46–1.85)	1.42 (1.26–1.60)	278 (9.80%)	1.62 (1.44–1.83)	1.64 (1.45–1.85)	1.62 (1.44–1.83)
Classification of Disease	Pulmonary TB	7585 (5.31%)	1.00	1.00	8215 (5.75%)	1.00	1.00	1.00
	EPTB	2324 (8.55%)	1.51 (1.44–1.58)	1.22 (1.16–1.28)	1786 (6.57%)	1.03 (0.97–1.08)	1.02 (0.97–1.08)	1.02 (0.97–1.08)
HIV status	Positive on ART	4056 (6.07%)	2.03 (1.90–2.17)	1.67 (1.58–1.78)	3435 (5.14%)	1.00 (0.95–1.06)	0.91 (0.86–0.96)	0.90 (0.85–0.95)
	Positive not on ART	2267 (8.35%)	2.88 (2.68–3.09)	2.51 (2.33–2.70)	2416 (7.90%)	1.59 (1.50–1.69)	1.57 (1.47–1.68)	1.52 (1.42–1.62)
	Positive ART status unknown	1243 (7.98%)	2.70 (2.50–2.93)	2.40 (2.21–2.61)	951 (6.10%)	1.21 (1.12–1.30)	1.23 (1.13–1.34)	1.19 (1.10–1.29)
	Negative	1155 (2.99%)	1.00	1.00	1971 (5.09%)	1.00	1.00	1.00
	HIV status unknown	1188 (5.47%)	1.86 (1.72–2.02)	1.77 (1.62–1.92)	1498 (6.90%)	1.37 (1.28–1.47)	1.43 (1.33–1.53)	1.40 (1.31–1.51)
Treatment supervision (Intensive Phase)	Yes	1514 (5.65%)	1.00	1.00	1509 (5.63%)	1.00	1.00	1.00
	No	7103 (5.92%)	1.06 (1.01–1.13)	0.78 (0.74–0.83)	7084 (5.91%)	1.07 (1.01–1.13)	0.79 (0.74–0.83)	0.80 (0.75–0.84)
	Missing	1292 (5.55%)	1.00 (0.93–1.08)	6.24 (4.91–7.92)	1408 (6.04%)	1.11 (1.03–1.19)	10.74 (8.00–14.40)	10.40 (7.75–13.95)
Treatment supervision (Continuation Phase)	Yes	76 (1.04%)	1.00	1.00	48 (0.66%)	1.00	1.00	1.00
	No	8541 (6.13%)	6.41 (5.11–8.03)	7.81 (6.20–9.84)	8545 (6.13%)	10.36 (7.78–13.73)	12.56 (9.42–14.40)	12.03 (9.02–16.04)
	Missing	1292 (5.55%)	5.83 (4.62–7.34)	(omitted)	1408 (6.04%)	10.26 (7.69–13.68)	(omitted)	(omitted)
**Overall**		**9909 (5.83%)**	**–**	**–**	**10001 (5.88%)**	**–**	**–**	

*EPTB* extra-pulmonary tuberculosis

^a^Sample excludes patients still on treatment at ≥10 months and patients with no available outcome (i.e. outcome not assigned)

^b^Sub-distribution hazard ratios from competing risk regression model proposed by Fine and Gray where death is considered a competing risk

^c^Bacteriologically confirmed includes Xpert MTB/RIF, smear, or culture (and if aspiration/biopsy or cerebral spinal fluid (CSF) was listed, although the corresponding laboratory method was unknown). Clinically diagnosed includes X-ray and tuberculin skin test

By age, the highest proportion of cases resulting in loss to follow up was observed among young adults (8.3%). Figure 2b shows the probability of retention in care over within 10 months by age group. The hazards of loss to follow-up was significantly elevated for young adults (aHR 1.43, 95% CI: 1.33, 1.54) compared to adults aged 25–49. Children, young adolescents and older adults had significantly lower hazards of loss to follow-up compared to adult patients.

Previous TB treatment was associated with death (aHR 1.23, 95% CI 1.13, 1.35) and loss to follow-up (aHR 1.74, 95% CI 1.61, 1.88) compared to new TB cases, as was unknown treatment history. Lack of treatment supervision during the continuation phase (as reported in ETR) had a strong association with both death (aHR 7.81, 95% CI 6.20, 9.84) and loss to follow-up (aHR 12.56, 95% CI 9.42, 14.40). Patients with extra-pulmonary TB had significantly greater hazards of death (aHR 1.22, 95% CI 1.16, 1.28) but not loss to follow-up (aHR 1.02, 95% CI 0.97, 1.08). Similarly, clinically diagnosis was significantly associated with death compared to bacteriological confirmation (aHR 1.36, 95% CI 1.28, 1.43), but clinical diagnosis was not associated with increased hazards of loss to follow-up (aHR 1.02, 95% CI 0.97, 1.09).

When compared to patients without HIV, patients with HIV on ART had slightly lower hazard of loss to follow-up (aHR 0.91, 95% CI: 0.86, 0.96) but greater hazard of death (aHR 1.67, 95% CI: 1.58, 1.78). Patients with HIV that were not on ART had over 1.5 times the hazard of loss to follow-up and more than twice the hazards of death when compared to patients without HIV. Patients with HIV but an unknown ART status and those with an unknown HIV status also had an increased hazards of loss to follow-up and death than those without HIV.

Compared to cases included in outcome analyses, a higher proportion of excluded patients (*n* = 12,873) were previously treated and had extra-pulmonary TB (Table 5). Furthermore, only 14.4% of excluded patients who were smear positive at initiation converted at two months compared to 70.9% of those included in the analysis, suggesting that excluded cases may have been more complicated patients who required extended treatment. Despite these differences, the sensitivity analyses for all-cause mortality and loss to follow-up including those still on treatment ≥10 months yielded similar findings to the primary models (Table 6). Similarly, the sub-distribution hazard ratios for loss to follow-up by age group were similar to the hazard ratios obtained in the primary and the sensitivity analysis, even when accounting for the competing risk of death.

**Table 5:** Comparison of participants who were still on treatment or transferred out (*n* = 12,873) compared to those included in the final outcomes analysis (*n* = 170,017)

Characteristics		Excluded^a^ *N* = 12873	Included *N* = 170017
Age at start of treatment (years)	<10 (children)	875 (6.80%)	17384 (10.22%)
	10–14 (young adolescents)	120 (0.93%)	1912 (1.12%)
	15–19 (older adolescents)	250 (1.94%)	3681 (2.17%)
	20–24 (young adults)	945 (7.34%)	10741 (6.32%)
	25–49 (adults)	9226 (71.67%)	114137 (67.13%)
	≥50 (older adults)	1457 (11.32%)	22162 (13.04%)
District in Gauteng	Ekurhuleni Metropolitan Municipality	4385 (34.06%)	59919 (35.24%)
	City of Johannesburg	8488 (65.94%)	110098 (64.76%)
Registration year	2010	3223 (25.04%)	29481 (17.34%)
	2011	2301 (16.29%)	29698 (17.47%)
	2012	2097 (16.29%)	28747 (16.91%)
	2013	2094 (16.27%)	28778 (16.93%)
	2014	1845 (14.33%)	27661 (16.27%)
	2015	1313 (10.20%)	25652 (15.09%)
Sex	Female	5953 (46.24%)	76388 (44.93%)
Case definition^b^	Bacteriologically confirmed	8720 (67.74%)	117640 (69.19%)
	Clinically diagnosed	2110 (16.39%)	27548 (16.20%)
	Missing/Unknown	2043 (15.87%)	24829 (14.60%)
Full case definition	Xpert MTB/RIF	1400 (10.88%)	27082 (15.93%)
	Smear microscopy	6377 (49.54%)	80815 (47.53%)
	Culture	429 (3.33%)	5209 (3.06%)
	Aspiration/biopsy	389 (3.02%)	3534 (2.08%)
	CSF	125 (0.97%)	1000 (0.59%)
	X-ray	1848 (14.36%)	21872 (12.86%)
	Skin Test	262 (2.04%)	5676 (3.34%)
	Unknown clinical diagnosis	2043 (15.87%)	24829 (14.60%)
Patient category	New Patient	11740 (91.20%)	160512 (94.41%)
	Previously treated	792	6667
	Relapse	534 (4.15%)	4693 (2.76%)
	Re-treatment after failure	91 (0.71%)	719 (0.42%)
	Re-treatment after LTFU (default)	167 (1.30%)	1255 (0.74%)
	Unknown previous TB treatment history	341 (2.65%)	2838 (1.67%)
Classification of Disease	Pulmonary TB	9877 (76.73%)	142843 (84.02%)
	EPTB	2996 (23.27%)	27174 (15.98%)
Smear status at initiation	Positive	4298 (33.39%)	63984 (37.63%)
	Negative	3261 (25.33%)	40547 (23.85%)
	Missing	5314 (41.28%)	65486 (38.52%)
HIV status	Positive, on ART	4550 (35.35%)	66871 (39.33%)
	Positive, not on ART	2608 (20.26%)	27149 (15.97%)
	Positive, ART status unknown	1565 (12.16%)	15583 (9.17%)
	Negative	2092 (16.25%)	38692 (22.76%)
	HIV status unknown	2058 (15.99%)	21722 (12.78%)
CD4 count (cells/mm^3^) if HIV positive^c^	≤100	2170 (16.86%)	23843 (14.02%)
	101–250	1539 (11.96%)	18895 (11.11%)
	251–350	441 (3.43%)	6244 (3.67%)
	351–500	326 (2.53%)	4551 (2.68%)
	>500	213 (1.65%)	3091 (1.82%)
	Missing	8184 (63.57%)	113433 (66.70%)
	Median (IQR)	126 (52–250)	113 (45–221)
Started on cotrimoxazole prophylaxis	Yes	6570 (75.32%)	86984 (79.36%)
	No	1237 (14.18%)	13535 (12.35%)
	Missing/unknown	916 (10.50%)	9084 (8.29%)
Treatment regimen	Regimen 1 (2 RHZE/4 RH)	11032 (85.70%)	146174 (85.98%)
	Regimen 2 (2 RHZES/1 HRZE/5 HRE)	1063 (8.26%)	8301 (4.88%)
	Regimen 3B (2 RHZE / 4 RH)	778 (6.04%)	15534 (9.14%)
	Other	0 (0.00%)	8 (0.00%)
Treatment supervision (Intensive Phase)	Yes	2356 (18.30%)	26814 (15.77%)
	No	9205 (71.51%)	119911 (70.53%)
	Missing	1312 (10.19%)	23292 (13.70%)
Treatment supervision (Continuation Phase)	Yes	414 (3.22%)	7308 (4.30%)
	No	11147 (86.59%)	139417 (82.00%)
	Missing	1312 (10.19%)	23292 (13.70%)
Smear conversion at 2 months^d^	Yes	185 (14.39%)	22291 (70.88%)
	No	1101 (85.61%)	9160 (29.12%)
	NA (Not smear positive at baseline or < 2 available smears)	8176	97894
Any smear conversion^e^	Yes	672 (36.60%)	55956 (95.06%)
	No	1164 (63.40%)	2905 (4.94%)
	NA (Not smear positive at baseline or < 2 available smears)	8176	97894

*Xpert MTB/RIF* GeneXpert MTB/RIF, *CSF* cerebral spinal fluid, *EPTB* extra-pulmonary tuberculosis, *LTFU* loss to follow-up, *HIV* human immunodeficiency virus, *ART* antiretroviral therapy, *IQR* interquartile range

^a^Excluded cases include those who were still on treatment at ten months, those who were lost to follow-up, and those with unknown outcomes

^b^Bacteriologically confirmed includes Xpert MTB/RIF, smear, or culture (and if aspiration/biopsy or cerebral spinal fluid (CSF) was listed, although the corresponding laboratory method was unknown). Clinically diagnosed includes X-ray and tuberculin skin test

^c^Reported for HIV positive patients only

^d^Smear conversion at two months is two consecutive negative smears at least 30 days apart within two months of treatment initiation. One smear is sent monthly for smear microscopy

^e^Any smear conversion is two consecutive negative smears at least 30 days apart at any time after treatment initiation. Ascertained at the end of treatment

**Table 6:** Cox proportional hazard models for all-cause mortality and loss to follow-up for all patients with an outcome assigned (*n* = 173,055^a^)

Characteristics		Proportion with outcome [Death]	Crude HR and 95% CI	Adjusted HR and 95% CI	Proportion with outcome [LTFU]	Crude HR and 95% CI	Adjusted HR and 95% CI	Adjusted sub-HR^b^ and 95% CI
Age at start of treatment	<10 (children)	209 (1.19%)	0.20 (0.18–0.23)	0.23 (0.20–0.26)	640 (3.64%)	0.60 (0.55–0.65)	0.63 (0.58–0.69)	0.64 (0.59–0.71)
	10–14 (young adolescents)	53 (2.71%)	0.45 (0.34–0.59)	0.48 (0.37–0.63)	60 (3.07%)	0.48 (0.38–0.62)	0.52 (0.40–0.67)	0.53 (0.41–0.69)
	15–19 (older adolescents)	110 (2.94%)	0.50 (0.42–0.61)	0.62 (0.52–0.75)	224 (5.99%)	0.98 (0.85–1.11)	1.05 (0.92–1.20)	1.07 (0.93–1.22)
	20–24 (young adults)	392 (3.60%)	0.62 (0.56–0.69)	0.72 (0.64–0.79)	896 (8.22%)	1.36 (1.27–1.46)	1.44 (1.34–1.54)	1.45 (1.35–1.55)
	25–49 (adults)	6745 (5.80%)	1.00	1.00	7082 (6.09%)	1.00	1.00	1.00
	≥50 (older adults)	2447 (10.84%)	1.92 (1.83–2.01)	2.12 (2.02–2.22)	1155 (5.11%)	0.87 (0.81–0.92)	0.85 (0.80–0.91)	0.82 (0.77–0.87)
Registration year	2010	1858 (6.49%)	1.02 (0.95–1.09)	0.99 (0.93–1.06)	1855 (6.18%)	1.12 (1.05–1.19)	1.04 (0.98–1.11)	1.04 (0.98–1.12)
	2011	1835 (6.07%)	1.00	1.00	1667 (5.52%)	1.00	1.00	1.00
	2012	1607 (5.50%)	0.91 (0.85–0.97)	0.91 (0.86–0.98)	1683 (5.76%)	1.05 (0.98–1.12)	1.08 (1.01–1.16)	1.08 (1.01–1.16)
	2013	1655 (5.65%)	0.94 (0.88–1.00)	0.96 (0.89–1.02)	1743 (5.95%)	1.09 (1.02–1.16)	1.18 (1.10–1.26)	1.17 (1.10–1.26)
	2014	1550 (5.50%)	0.92 (0.86–0.98)	0.98 (0.91–1.06)	1604 (5.70%)	1.05 (0.98–1.13)	1.20 (1.11–1.29)	1.19 (1.11–1.29)
	2015	1451 (5.55%)	0.93 (0.87–0.99)	1.05 (0.96–1.15)	1505 (5.76%)	1.06 (0.99–1.14)	1.23 (1.13–1.34)	1.23 (1.12–1.34)
Sex	Female	4521 (5.82%)	1.01 (0.97–1.05)	1.04 (1.00–1.08)	3913 (5.03%)	0.77 (0.74–0.81)	0.77 (0.74–0.81)	0.77 (0.74–0.81)
	Male	5435 (5.70%)	1.00	1.00	6144 (6.45%)	1.00	1.00	1.00
Case definition^c^	Bacteriologically confirmed	6588 (5.52%)	1.00	1.00	7162 (6.00%)	1.00	1.00	1.00
	Clinically diagnosed	1711 (6.08%)	1.08 (1.03–1.14)	1.34 (1.27–1.42)	1515 (5.38%)	0.88 (0.83–0.93)	1.01 (0.95–1.07)	1.00 (0.94–1.06)
	Missing/unknown	1657 (6.48%)	1.15 (1.09–1.21)	1.49 (1.41–1.58)	1380 (5.40%)	0.87 (0.82–0.92)	1.02 (0.96–1.09)	1.01 (0.95–1.07)
Patient category	New Patient	9162 (5.61%)	1.00	1.00	9073 (5.56%)	1.00	1.00	1.00
	Previously treated	515 (7.47%)	1.27 (1.16–1.39)	1.20 (1.10–1.32)	705 (10.22%)	0.72 (1.59–1.85)	1.70 (1.57–1.84)	1.71 (1.58–1.85)
	Unknown TB treatment history	279 (9.53%)	1.62 (1.44–1.83)	1.40 (1.24–1.57)	279 (9.53%)	1.60 (1.42–1.80)	1.62 (1.43–1.82)	1.61 (1.42–1.81)
Classification of Disease	Pulmonary TB	7615 (5.26%)	1.00	1.00	8259 (5.71%)	1.00	1.00	1.00
	EPTB	2341 (8.26%)	1.46 (1.40–1.53)	1.19 (1.13–1.25)	1798 (6.34%)	1.00 (0.95–1.05)	1.00 (0.94–1.05)	1.00 (0.94–1.06)
HIV status	Positive on ART	4074 (5.98%)	2.01 (1.89–2.15)	1.66 (1.56–1.78)	3452 (5.07%)	1.00 (0.94–1.05)	0.91 (0.86–0.96)	0.80 (0.85–0.95)
	Positive not on ART	2277 (8.24%)	2.86 (2.67–3.07)	2.50 (2.32–2.69)	2156 (7.80%)	1.59 (1.49–1.69)	1.57 (1.47–1.67)	1.51 (1.42–1.62)
	Positive ART status unknown	1247 (7.80%)	2.65 (2.45–2.87)	2.36 (2.17–2.57)	954 (5.97%)	1.18 (1.10–1.28)	1.21 (1.12–1.31)	1.18 (1.09–1.28)
	Negative	1162 (2.96%)	1.00	1.00	1983 (5.05%)	1.00	1.00	1.00
	HIV status unknown	1196 (5.42%)	1.85 (1.71–2.01)	1.79 (1.62–1.91)	1512 (6.85%)	1.37 (1.28–1.47)	1.42 (1.33–1.53)	1.40 (1.30–1.50)
Treatment supervision (Intensive Phase)	Yes	1518 (5.49%)	1.00	1.00	1516 (5.48%)	1.00	1.00	1.00
	No	7139 (5.87%)	1.09 (1.03–1.15)	0.80 (0.76–0.85)	7124 (5.86%)	1.10 (1.04–1.16)	0.80 (0.76–0.85)	0.81 (0.76–0.86)
	Missing	1299 (5.45%)	1.01 (0.94–1.09)	6.45 (5.08–8.19)	1417 (5.95%)	1.12 (1.04–1.20)	11.17 (8.33–14.99)	10.69 (7.97–14.34)
Treatment supervision (Continuation Phase)	Yes	77 (0.99%)	1.00	1.00	48 (0.63%)	1.00	1.00	1.00
	No	8581 (6.06%)	6.66 (5.31–8.35)	8.03 (6.37–10.12)	8592 (6.07%)	10.75 (8.10–14.28)	12.98 (9.73–17.31)	12.36 (9.26–16.48)
	Missing	1299 (5.45%)	5.99 (4.75–7.55)	(omitted)	1417 (5.95%)	10.54 (7.91–14.06)	(omitted)	(omitted)
Overall		**9956 (5.75%)**	**–**	**–**	**10057 (5.81%)**	**–**	**–**	

*EPTB* extra-pulmonary tuberculosis

^a^Sample excludes patients with no available outcome (i.e. transferred out, outcome not assigned)

^b^Sub-distribution hazard ratios from competing risk regression model proposed by Fine and Gray where death is considered a competing risk

^c^Bacteriologically confirmed includes Xpert MTB/RIF, smear, or culture (and if aspiration/biopsy or cerebral spinal fluid (CSF) was listed, although the corresponding laboratory method was unknown). Clinically diagnosed includes X-ray and tuberculin skin test

## Discussion

Among persons treated for TB in the City of Johannesburg and Ekurhuleni Metropolitan Municipality between 2010 and 2015, we found that a successful clinical outcome was achieved in excess of 80% in all age groups. All-cause mortality increased with age, loss to follow-up was greater in older adolescents than younger adolescents, and young adults ages 20–24 had the highest rates of loss to follow-up. Furthermore, young adults with HIV were less likely to be on ART than other age groups. Our findings indicate that older adolescents and young adults are a key group to target for interventions to improve retention, thus improving success rates.

While previous evidence on TB outcomes across age groups is not definitive, others have observed poor outcomes in older adolescents and young adults in South Africa [24]. A similar study in Cape Town from 2009 to 2013 also identified greater treatment success in young adolescents compared to older adolescents and young adults and that both TB deaths and loss to follow-up increase with age. A study from the Western Cape using ETR data from 2011 reported that older adolescents and young adults were at greatest risk of treatment non-completion, especially among those living with HIV [11]. Despite numerous calls to prioritize research aimed at improving clinical outcomes in these age groups [1, 25], little progress has been made. A systematic review of barriers to TB treatment initiation in sub-Saharan Africa found that the experiences of children and youth were not well described and the authors did not identify any studies exploring loss to follow up as a function of age [26]. Clearly, much work must be done in this age group in order to understand the best approaches to retain adolescents and young adults in care and promote treatment success.

Nearly two-thirds (65%) of our study population was HIV co-infected, which falls within the range of the national estimates for 2015 (59%) and those observed from Johannesburg alone (71%) [14, 27]. HIV is a well-known risk factor for TB mortality, especially among those who are not on ART [2, 3]. Accordingly, we observed increased hazards of death in patients with HIV who were not on treatment. While South African guidelines recommend ART initiation in all HIV/TB co-infected patients, there was a lower percentage (71%) of ART coverage among persons with complete HIV and ART data in our study than that previously reported at the provincial level (90%). This discrepancy may be influenced in part by the extent of missing data in our study or that our study is limited to the metropolitan municipalities of the province. Additionally, people living with HIV who were not on ART had higher hazards of LTFU. There are inferable mechanisms for how ART might improve retention in TB care: co-infected patients on ART may have greater health-seeking behaviour or care and follow up for TB may be more intensive among those also receiving ART. If being on ART can improve LTFU, this may further support early initiation of ART through test-and-treat approaches. We also observed that ART coverage was lowest among young adults (54%), further evidencing an important service delivery gap in this age group. Increasing ART coverage is important for both preventing TB and improving TB outcomes [28], and collaborative TB/HIV activities have been recommended as a strategy for national programs [29]. However significant barriers to scale up of these activities persist in South Africa [30–33]. For example, routine provision of isoniazid prophylaxis in people living with HIV and rapid initiation of ART among co-infected persons have been identified as the least available TB/HIV services in public medical facilities [33].

While we observed greater all-cause mortality in older age groups, it is possible some deaths may have been attributable causes other than TB (e.g. poorer underlying health, untreated comorbid conditions etc.). On the contrary, older people with TB may have more complex TB disease with longer delays to diagnosis (as sputum smears have fewer bacilli, and patients exhibit a lower frequency of fever and haemoptysis) and often suffer from more adverse events and comorbidities that may affect treatment outcomes, including death [34]. Older patients also have a higher probability of treatment failure and of mortality due to tuberculosis [35]. For these older age groups, more aggressive screening and management of comorbid conditions and routine screening of kidney and liver function to ensure appropriate dosing in this population may help to improve treatment outcomes. Nonetheless, frequent monitoring of the TB burden in older age groups, especially in the subset of older people living with HIV, is warranted to allow health policy makers and care providers to plan for meeting the needs of this population [35].

We also observed differences in treatment outcome by sex and TB disease characteristics. First, women in our study had lower hazards of LTFU than men. It has long been known that women tend to adhere better and complete TB treatment more often than men. While the exact reasons are still unknown, this phenomenon has been documented across settings [36–38]. Second, patients with EPTB had greater hazards of death. While EPTB is more often seen in young children, young children in our study had the lowest hazards of death, thus EPTB is likely not merely a proxy for age. Rather, this may represent delayed diagnosis or greater severity or more advanced disease from co-morbid conditions such as HIV. Lastly, previous TB treatment was associated with both death and LTFU. It is possible that retreatment cases have more extensive lung damage, undetected drug resistant, or comorbidities resulting from a previous TB episode, such as chronic pulmonary obstructive disorder, that could affect outcome of subsequent TB treatments.

Our study has a number of limitations. Issues related to the accuracy of completeness of national data are well described [13, 39–41]. While data on sex and age are highly reliable, data on HIV status, ART status, and treatment outcome require improvement. Earlier version of ETR did not have provision for Xpert MTB/RIF results and cases were entered under smear microscopy. Data used in this study were retrospective, routinely collected, and de-identified. Due to this, we were unable to verify HIV status, ART use, CD4 counts or pursue validation of bacteriologic confirmation via the National Health Laboratory Service. Similarly, we were not able to link TB programmatic outcome data to vital statistics registers or accurately determine mortality due to TB. It is possible that some patients considered as lost to follow up actually died and are thus misclassified in the present analysis. As noted in previous analyses, the ETR data were subject to missingness [13], particularly in records of treatment supervision. Since clinic staff may have made errors in the transcription of treatment supervision or had different understandings of how supervision was defined, we are cautious about over-interpretation of this variable. Finally, patients who were still in care at 10 months, transferred out, or were missing an outcome were excluded from the outcome analysis, which may have introduced selection bias as the excluded patients appeared have more severe cases on TB. However, a sensitivity analysis including patients still on treatment yielded similar results.

## Conclusions

Results from this study provide important insight for the planning and implementation of TB and HIV activities and tailoring of health services to the needs of different age and risk groups. Young adults in urban areas of Gauteng Province experience a disproportionate burden of LTFU and low coverage of ART among co-infected patients. This group should be targeted for interventions aimed at improving clinical outcomes and retention in both TB and HIV care. In addition, as people with HIV in low- and middle-income countries who start treatment early experience near-normal life expectancy, health care systems need to prepare for the increase of TB in aging populations that are more difficult to treat and have worse outcomes.

## Data Availability

The datasets generated and/or analysed during the current study are not publicly available as the data are owned by the National Department of Health (South Africa) and governed by the Human Research Ethics Committee (University of the Witwatersrand, Johannesburg, South Africa). All relevant data are included in the paper. The full data are available from the Health Economics and Epidemiology Research Office for researchers who meet the criteria for access to confidential data and with permission from the owners of the data. Contact the organization at information@heroza.org for additional information regarding data access.

## Abbreviations

aHR: adjusted hazards ratio TB- tuberculosis
ART: Antiretroviral therapy
CSF: Cerebral spinal fluid
EPTB: Extra-pulmonary tuberculosis
ETR: South Africa’s National Electronic TB Register
HIV: Human immunodeficiency virus
HR: Hazard ratio
IQR: Interquartile range
LTFU: Loss to follow-up
MTB: *Mycobacterium tuberculosis*
NHLS: National Health Laboratory Services
PTB: Pulmonary tuberculosis
RIF: Rifampicin
sHR: sub-distribution hazard ratio
TST: Tuberculin skin test
